# The chromosomal genome sequence of the fragile freshwater sponge, *Eunapius fragilis *(Leidy, 1851) and its associated microbial metagenome sequences

**DOI:** 10.12688/wellcomeopenres.24165.1

**Published:** 2025-05-23

**Authors:** Sally P. Leys, Ute Hentschel, Cole Gregory Easson, Damon Stimson, Jose Victor Lopez, Graeme Oatley, Elizabeth Sinclair, Eerik Aunin, Noah Gettle, Camilla Santos, Michael Paulini, Haoyu Niu, Victoria McKenna, Rebecca O’Brien, Wellcome Sanger Institute Tree of Life Management, Samples and Laboratory Team, Wellcome Sanger Institute Scientific Operations: Sequencing Operations, Wellcome Sanger Institute Tree of Life Core Informatics Team, EBI Aquatic Symbiosis Genomics Data Portal Team, Aquatic Symbiosis Genomics Project Leadership

**Affiliations:** 1 University of Alberta Department of Biological Sciences, Edmonton, Alberta, Canada; 2 GEOMAR Helmholtz Centre for Ocean Research Kiel, Kiel, Germany; 3 Middle Tennessee State University Department of Biology, Murfreesboro, Tennessee, USA; 4 National Coral Reef Institute, Nova Southeastern University, Fort Lauderdale, Florida, USA; 5 Wellcome Sanger Institute, Hinxton, England, UK

**Keywords:** Eunapius fragilis, fragile freshwater sponge, genome sequence, chromosomal, Spongillida, microbial metagenome

## Abstract

We present a genome assembly from a specimen of *Eunapius fragilis* (fragile freshwater sponge; Porifera; Demospongiae; Spongillida; Spongillidae). The genome sequence has a total length of 218.91 megabases. Most of the assembly (99.98%) is scaffolded into 23 chromosomal pseudomolecules. The mitochondrial genome has also been assembled and is 28.5 kilobases in length. Gene annotation of this assembly on Ensembl identified 26,614 protein-coding genes. Additionally, eight high-quality bacterial metagenomes belonging to the *Bacteroidota* and *Pseudomonadota* phyla were assembled.

## Species taxonomy

Eukaryota; Opisthokonta; Metazoa; Porifera; Demospongiae; Heteroscleromorpha; Spongillida; Spongillidae; *Eunapius*; *Eunapius fragilis* (Leidy, 1851) (NCBI:txid91661)

## Background

*Eunapius fragilis* is one of 19 described species in the genus *Eunapius* (family Spongillidae), a monophyletic group of freshwater sponges that includes only eight families, of which Spongillidae and Metaniidae are the most widely distributed ( de Voogd *et al.*, 2024; Manconi & Pronzato, 2008). First described as *Spongilla fragilis* by Leidy in 1851 from specimens found on submerged stones in the Delaware and Schuylkill Rivers, *E. fragilis* is considered the first freshwater sponge formally described in North America ( Leidy, 1851). It has since been reported from across Europe, Asia, North America, and southern Australia ( Copeland *et al.*, 2019; Muricy *et al.*, 2011; Penney & Racek, 1968).

*Eunapius fragilis* typically forms thin, dull tan to grey encrustations on submerged surfaces such as rocks and woody debris in both lentic and lotic habitats, but may appear olive-green when exposed to light due to the presence of algal symbionts, commonly dinoflagellates of the family Chlorellaceae ( Hall *et al.*, 2021; Manconi & Pronzato, 2016; Pröschold & Darienko, 2020). Morphological identification requires examination of the gemmoscleres – straight to slightly curved strongyles or oxeas bearing conspicuous spines – since the smooth megascleres (oxea) do not reliably distinguish it from co-occuring species such as *Ephydatia fluviatilis* ( Copeland *et al.*, 2019; Manconi & Pronzato, 2016).

Asexual reproduction via gemmules – resting bodies that encase totipotent cells within a thickened wall – is central to the ability of freshwater sponges to persist in dynamic or ephemeral aquatic environments ( Manconi & Pronzato, 2008; Wells *et al.*, 1938). These structures enable survival during drought, freezing, or other adverse conditions, and also facilitate dispersal, either by ingestion or by attachment to the appendages of motile vertebrates ( Manconi & Pronzato, 2008). In *E. fragilis*, gemmules form a distinct pavement layer covered by a common coat. Their micropyle openings, through which the cells emerge when conditions become favourable, typically face upwards—a feature that contrasts with the downward-facing micropyles observed in the closely related species *E. mackayi* ( Ricciardi & Reiswig, 1993).

Gemmules of *E. fragilis* tolerate a wide range of salinities and have been shown to survive for up to 27 days in 30 ppt seawater. Exposure to high salinity can induce a deep diapause, from which they may later recover upon cold treatment ( Fell, 1987; Fell, 1992). Their robustness has also facilitated laboratory hatching, making *E. fragilis* a promising model for studies in development, physiology, and cell signalling ( Elliott & Leys, 2007; Windsor & Leys, 2010; Windsor Reid *et al.*, 2018).

Sexual reproduction also occurs in this species, with larvae containing differentiated tissues including choanoderm and endopinacoderm. In Canada, larval stages have been reported in July, and in Japan during August ( Harrison & Cowden, 1975; Mukai, 1989; Windsor Reid *et al.*, 2018). Microbial symbioses in *E. fragilis* include green algae and diverse bacteria from the phyla Proteobacteria, Actinobacteria, Bacteroidetes and Firmicutes, with some strains investigated for secondary metabolite production ( Lo Giudice & Rizzo, 2023).

Despite the widespread distribution and ecological resilience of *E. fragilis*, detailed knowledge of the physiology, population structure, and ecological role of this species remains limited. The genome presented here is based on a specimen collected in O’Connor Lake on Vancouver Island, British Columbia in Canada, and hatched on a sterile Petri dish. RNA sequencing was performed on a wild specimen collected from a lentic stretch of Stewart’s Creek in the Stones River Watershed, Tennessee, USA, and the raw data is made available on sequence databases. The data provide a new resource for investigating sponge evolution, symbiosis, and environmental adaptation.

## Genome sequence report

**Figure 1.**
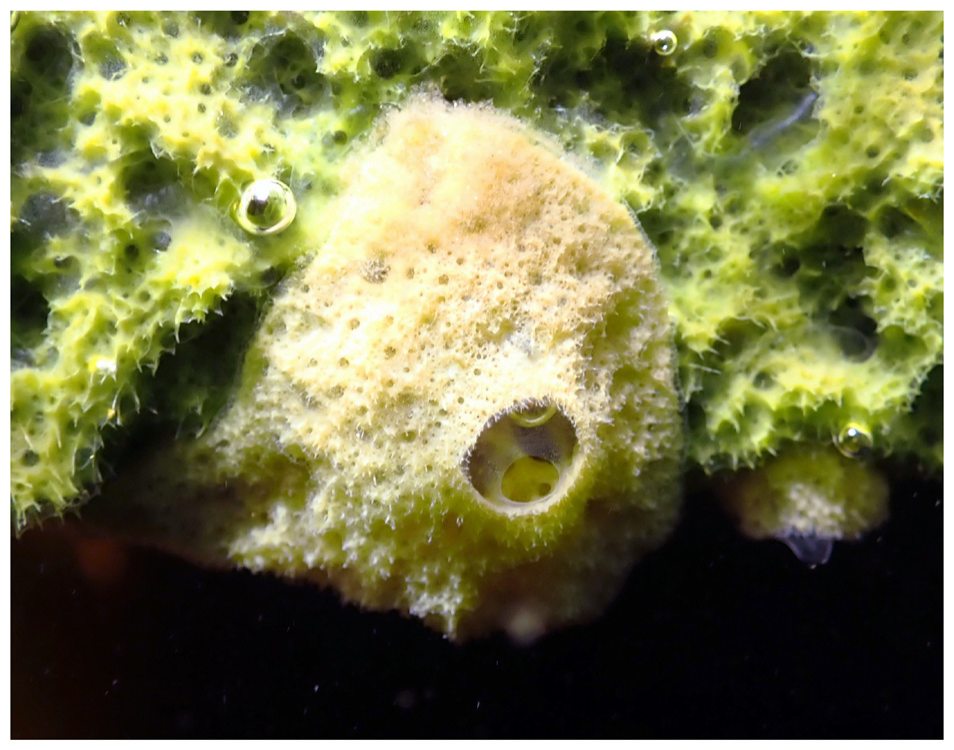
A representative wild specimen of *Eunapius fragilis*, surrounded by an *Ephydatia* sponge. The bubbles are probably due to the production of oxygen by algal symbionts. (Photograph by Sally Leys, 2024).

The genome of a specimen of *Eunapius fragilis* ( Figure 1) was sequenced using Pacific Biosciences single-molecule HiFi long reads, generating 184.53 Gb (gigabases) from 18.74 million reads. Based on the estimated genome size, the sequencing data provided approximately 118.0x coverage of the genome. Chromosome conformation Hi-C data produced 152.44 Gb from 1,009.51 million reads. RNA data from a different specimen was also generated for use in annotation (see methods). Specimen and sequencing details are provided in Table 1.

**Table 1.** Specimen and sequencing data for *Eunapius fragilis*.

Project information			
**Study title**	Eunapius fragilis		
**Umbrella BioProject**	PRJEB63655		
**Species**	*Eunapius fragilis*		
**BioSpecimen**	SAMEA8580183		
**NCBI taxonomy ID**	91661		
Specimen information			
**Technology**	**ToLID**	**BioSample accession**	**Organism part**
**PacBio long read sequencing**	odEunFrag1	SAMEA8580197	Somatic animal tissue
**Hi-C sequencing**	odEunFrag1	SAMEA8580200	Somatic animal tissue
**RNA sequencing**	odEunFrag2	SAMEA12922104	Somatic animal tissue
Sequencing information			
**Platform**	**Run accession**	**Read count**	**Base count (Gb)**
**Hi-C Illumina NovaSeq 6000**	ERR11641156	1.01e+09	152.44
**PacBio Sequel IIe**	ERR11641081	8.52e+05	5.48
**PacBio Sequel IIe**	ERR11641080	4.67e+06	56.74
**PacBio Sequel IIe**	ERR11641082	1.26e+06	16.18
**PacBio Sequel IIe**	ERR11641083	1.38e+06	7.96
**PacBio Revio**	ERR14747657	6.92e+06	61.28
**PacBio Revio**	ERR14749922	3.67e+06	36.89
**RNA Illumina NovaSeq 6000**	ERR11641155	5.71e+07	8.62

The primary haplotype was assembled, and contigs corresponding to an alternate haplotype were also deposited in INSDC databases. The assembly was improved by manual curation, which corrected 212 misjoins or missing joins and removed 8 haplotypic duplications. These interventions reduced the total assembly length by 47.54%, decreased the scaffold count by 99.58%, and increased the scaffold N50 by 623.84%. The final assembly has a total length of 218.91 Mb in 23 scaffolds, with 802 gaps, and a scaffold N50 of 9.93 Mb ( Table 2).

**Table 2.** Genome assembly data for *Eunapius fragilis*.

Genome assembly	
Assembly name	odEunFrag1.1
Assembly accession	GCA_963681505.1
*Alternate haplotype accession*	*GCA_963681495.1*
Assembly level for primary assembly	chromosome
Span (Mb)	218.91
Number of contigs	825
Number of scaffolds	23
Longest scaffold (Mb)	24.99
Assembly metric	Measure
Contig N50 length	0.49 Mb
Scaffold N50 length	9.93 Mb
Consensus quality (QV)	Primary: 60.4; alternate: 60.5; combined 60.5
*k*-mer completeness	Primary: 55.49%; alternate: 43.92%; combined: 71.17%
BUSCO *	C:60.0%[S:58.8%,D:1.2%],F:8.0%,M:32.1%,n:954
Percentage of assembly mapped to chromosomes	99.98%
Organelles	Mitochondrial genome: 28.5 kb
Genome annotation of assembly GCA_963681505.1 at Ensembl	
Number of protein-coding genes	26,614
Number of non-coding genes	962
Number of gene transcripts	37,297

* BUSCO scores based on the metazoa_odb10 BUSCO set using version 5.5.0. C = complete [S = single copy, D = duplicated], F = fragmented, M = missing, n = number of orthologues in comparison.

**Figure 2.**
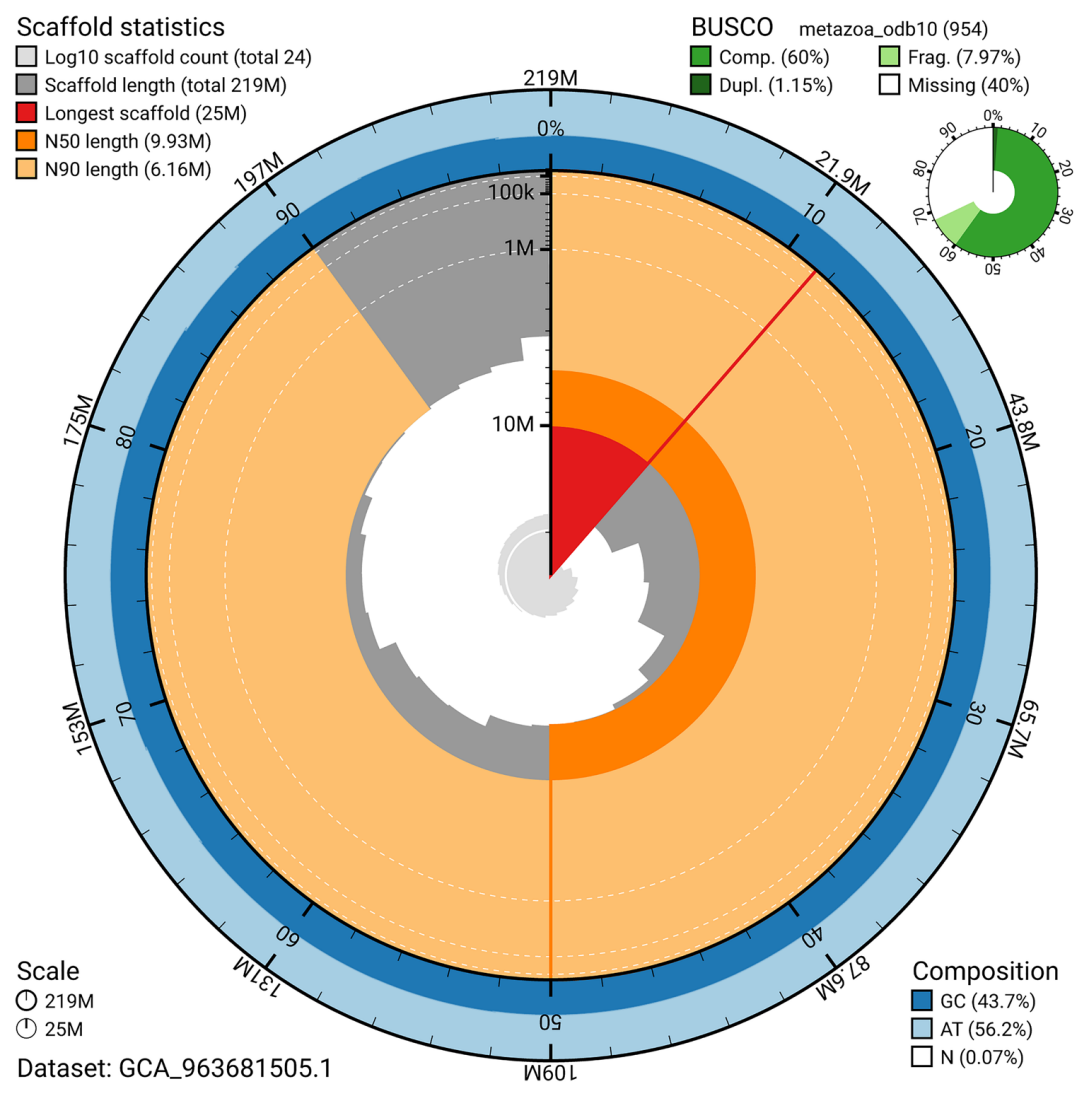
Genome assembly of *Eunapius fragilis*, odEunFrag1.1: metrics. The BlobToolKit snail plot provides an overview of assembly metrics and BUSCO gene completeness. The circumference represents the length of the whole genome sequence, and the main plot is divided into 1,000 bins around the circumference. The outermost blue tracks display the distribution of GC, AT, and N percentages across the bins. Scaffolds are arranged clockwise from longest to shortest and are depicted in dark grey. The longest scaffold is indicated by the red arc, and the deeper orange and pale orange arcs represent the N50 and N90 lengths. A light grey spiral at the centre shows the cumulative scaffold count on a logarithmic scale. A summary of complete, fragmented, duplicated, and missing BUSCO genes in the metazoa_odb10 set is presented at the top right. An interactive version of this figure is available at https://blobtoolkit.genomehubs.org/view/GCA_963681505.1/dataset/GCA_963681505.1/snail.

**Figure 3.**
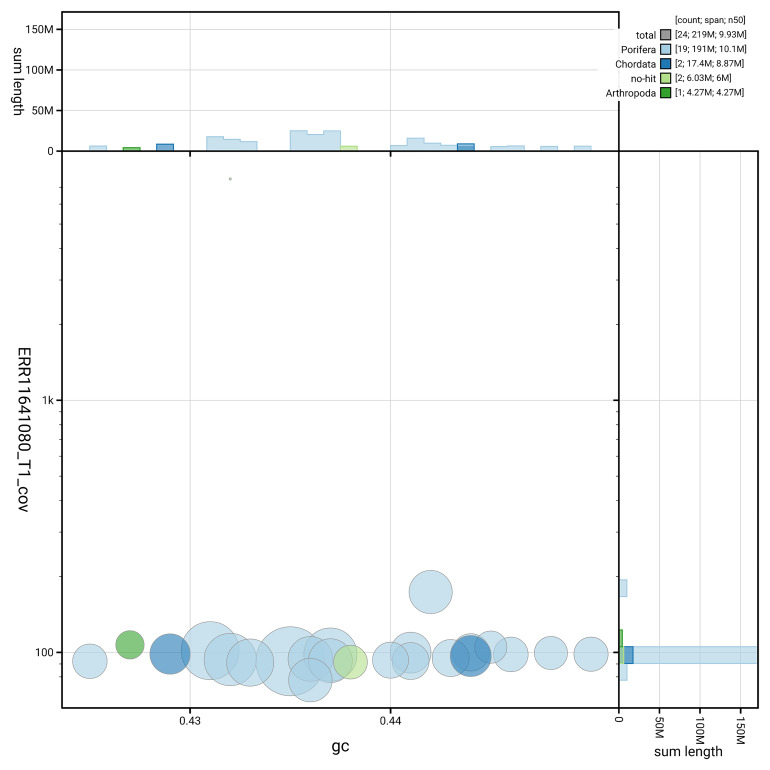
Genome assembly of *Eunapius fragilis*, odEunFrag1.1: BlobToolKit GC-coverage plot. Blob plot showing sequence coverage (vertical axis) and GC content (horizontal axis). The circles represent scaffolds, with the size proportional to scaffold length and the colour representing phylum membership. The histograms along the axes display the total length of sequences distributed across different levels of coverage and GC content. An interactive version of this figure is available at https://blobtoolkit.genomehubs.org/view/GCA_963681505.1/dataset/GCA_963681505.1/blob.

**Figure 4.**
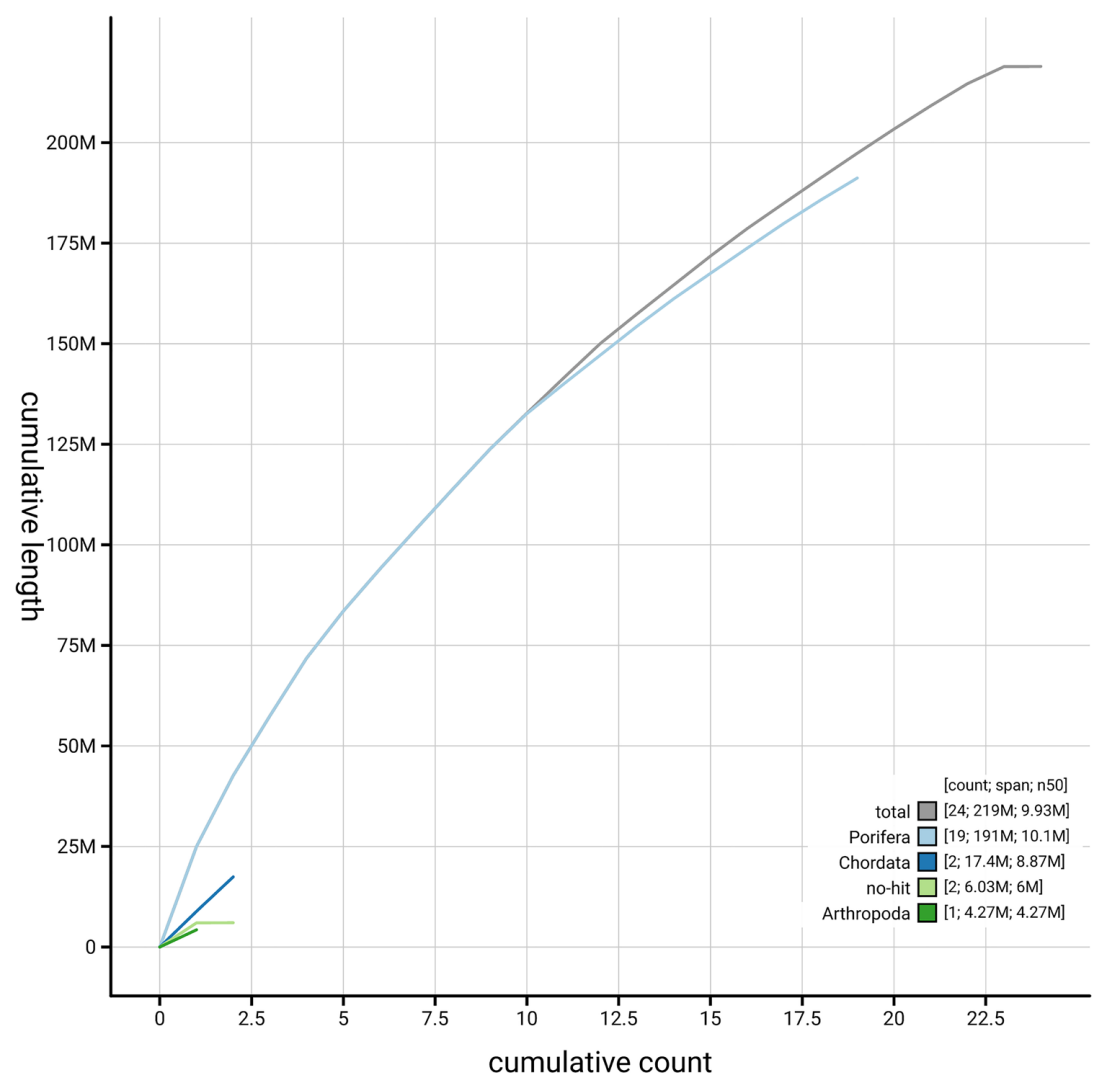
Genome assembly of *Eunapius fragilis,* odEunFrag1.1: BlobToolKit cumulative sequence plot. The grey line shows cumulative length for all scaffolds. Coloured lines show cumulative lengths of scaffolds assigned to each phylum using the buscogenes taxrule. An interactive version of this figure is available at https://blobtoolkit.genomehubs.org/view/GCA_963681505.1/dataset/GCA_963681505.1/cumulative.

The snail plot in Figure 2 provides a summary of the assembly statistics, indicating the distribution of scaffold lengths and other assembly metrics. Figure 3 shows the distribution of scaffolds by GC proportion and coverage. Figure 4 presents a cumulative assembly plot, with separate curves representing different scaffold subsets assigned to various phyla, illustrating the completeness of the assembly.

**Figure 5.**
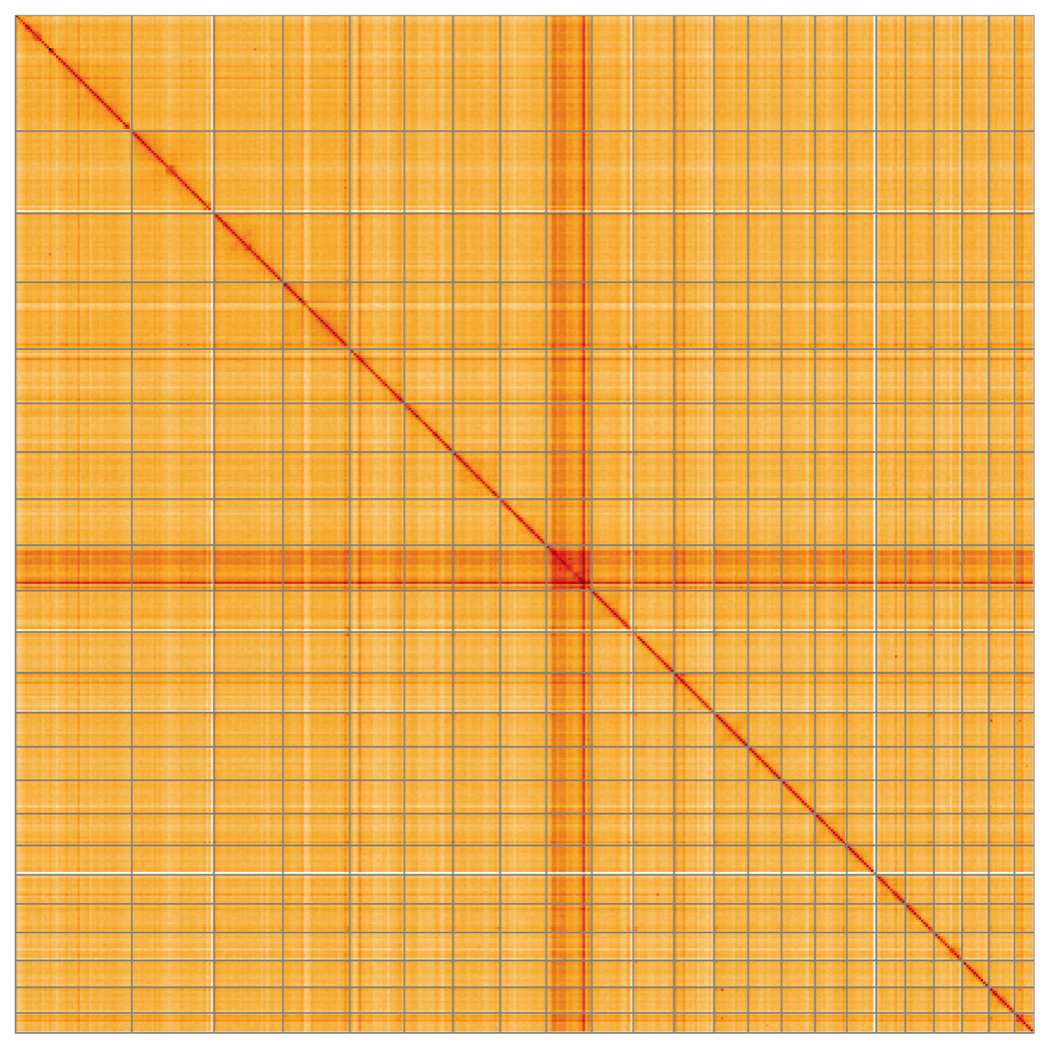
Genome assembly of *Eunapius fragilis*: Hi-C contact map of the odEunFrag1.1 assembly, visualised using HiGlass. Chromosomes are shown in order of size from left to right and top to bottom. An interactive version of this figure may be viewed at https://genome-note-higlass.tol.sanger.ac.uk/l/?d=U9e-c7MuTv-px9_nuJLqsQ.

Most of the assembly sequence (99.98%) was assigned to 23 chromosomal-level scaffolds. These chromosome-level scaffolds, confirmed by Hi-C data, are named according to size ( Figure 5; Table 3).

**Table 3.** Chromosomal pseudomolecules in the genome assembly of *Eunapius fragilis*, odEunFrag1.

INSDC accession	Name	Length (Mb)	GC%
OY811971.1	1	24.99	43.5
OY811972.1	2	17.64	43
OY811973.1	3	14.86	43.5
OY811974.1	4	14.35	43
OY811975.1	5	11.69	43.5
OY811976.1	6	10.49	43.5
OY811977.1	7	10.09	43.5
OY811978.1	8	9.93	43.5
OY811979.1	9	9.79	44
OY811980.1	10	8.87	44.5
OY811981.1	11	8.78	44
OY811982.1	12	8.57	43
OY811983.1	13	7.35	44.5
OY811984.1	14	7.2	44.5
OY811985.1	15	7.18	44
OY811986.1	16	6.83	44
OY811987.1	17	6.31	44.5
OY811988.1	18	6.26	42.5
OY811989.1	19	6.16	45
OY811990.1	20	6.0	44
OY811991.1	21	5.79	45
OY811992.1	22	5.5	44.5
OY811993.1	23	4.27	42.5
OY811994.1	MT	0.03	43

The mitochondrial genome was also assembled. This sequence is included as a contig in the multifasta file of the genome submission and as a standalone record in GenBank.

The host primary haplotype has a QV of 60.4, and the combined primary and alternate assemblies achieve an estimated QV of 60.5. BUSCO v.5.5.0 analysis of the host genome assembly using the metazoa_odb10 reference set ( *n* = 954) indicated a completeness score of 60% (single = 58.8%, duplicated = 1.2%).

## Genome annotation report

The *Eunapius fragilis* host genome assembly (GCA_963681505.1) was annotated at the European Bioinformatics Institute (EBI) on Ensembl Rapid Release. The resulting annotation includes 37,297 transcribed mRNAs from 26,614 protein-coding and 962 non-coding genes ( Table 2; https://rapid.ensembl.org/Eunapius_fragilis_GCA_963681505.1/Info/Index). The average transcript length is 4,670.88. There are 1.35 coding transcripts per gene and 5.69 exons per transcript. An alternative annotation for this genome is available here: https://github.com/Aquatic-Symbiosis-Genomics-Project/sponge_annotations/tree/main/results/odEunFrag1.

## Metagenome report

**Figure 6.**
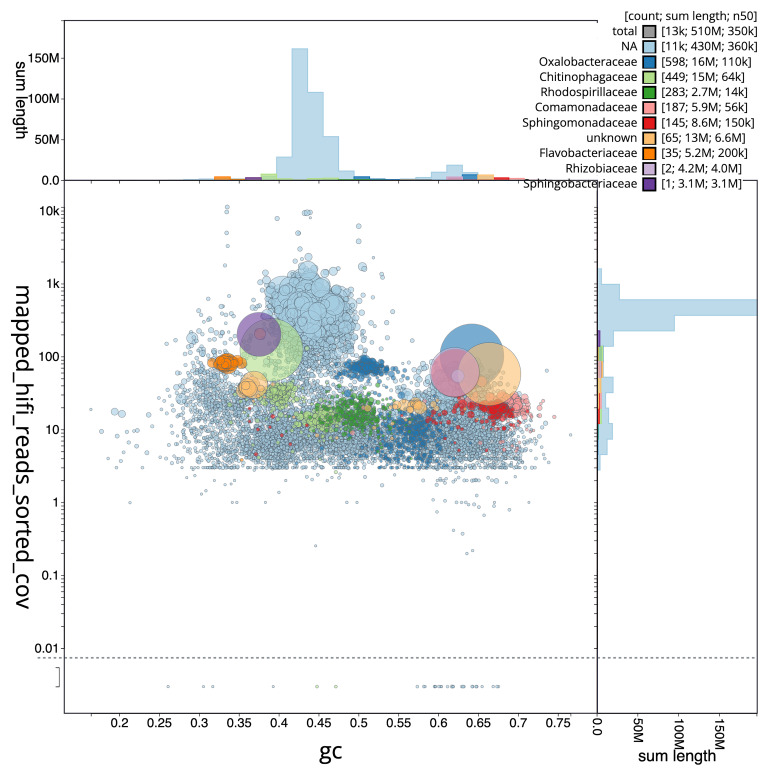
Blob plot of base coverage in mapped against GC proportion for sequences in the *Eunapius fragilis* metagenome. Binned contigs are coloured by family. Circles are sized in proportion to sequence length on a square root scale, ranging from 510 to 6,727,616. Histograms show the distribution of sequence length sum along each axis. An interactive version may be viewed here.

**Figure 7.**
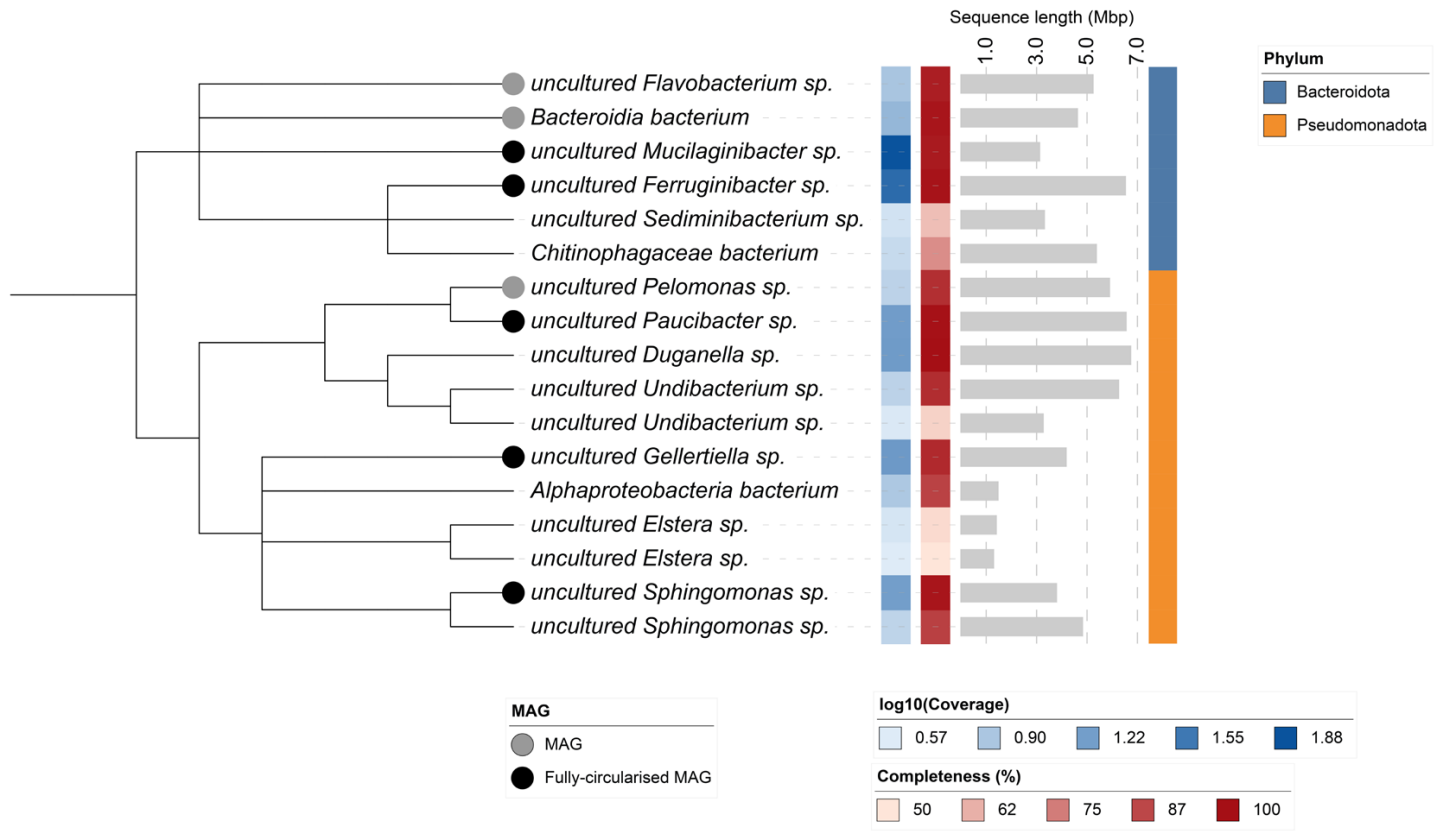
Cladogram showing the taxonomic placement of metagenome bins, constructed using NCBI taxonomic identifiers with **taxonomizr** and annotated in iTOL. The colour bar on the right indicates the phylum-level taxonomy. Additional tracks show sequencing coverage (log ^10^), percentage completeness and estimated genome size (Mbp). Bins that meet the criteria for MAGs are marked with a grey circle; those that are fully circularised MAGs are marked in black.

Seventeen binned genomes were generated from the metagenome assembly ( Figure 6) of which 8 were classified as high-quality metagenome assembled genomes (MAGs) (see methods). The completeness values for these assemblies range from approximately 50% to 100% with contamination below 5%. A cladogram of the binned metagenomes is shown in Figure 7. For details on binned genomes see Table 4.

**Table 4.** Quality metrics and taxonomic assignments of the binned metagenomes.

NCBI taxon	Taxid	GTDB taxonomy	Quality	Size (bp)	Contigs	Circular	Mean coverage	Completeness (%)	Contamination (%)
uncultured Duganella sp.	206097	s__Duganella sp000335815	Medium	6,727,617	1	Yes	18.03	100.0	0.53
uncultured Undibacterium sp.	686278	g__Undibacterium	Medium	6,254,173	193	No	6.23	93.2	3.63
Alphaproteobacteria bacterium	1913988	g__UBA6156	Medium	1,468,191	33	No	7.58	88.38	1.68
uncultured Sphingomonas sp.	158754	s__Sphingomonas sp005503355	Medium	4,823,420	144	No	5.96	88.89	4.87
Chitinophagaceae bacterium	1869212	g__JACMLD01	Medium	5,374,553	223	No	5.21	71.14	0.25
uncultured Sediminibacterium sp.	796894	g__Sediminibacterium	Medium	3,310,950	225	No	4.44	59.30	2.09
uncultured Elstera sp.	1662499	f__Elsteraceae	Medium	1,292,549	182	No	3.75	50.20	2.69
uncultured Undibacterium sp.	686278	g__Undibacterium	Medium	3,258,431	404	No	3.96	54.97	4.92
uncultured Elstera sp.	1662499	f__Elsteraceae	Medium	1,401,266	101	No	4.31	52.84	0.22
uncultured Paucibacter sp.	452440	g__Paucibacter	High	6,550,682	1	Yes	17.23	99.77	0.70
uncultured Gellertiella sp.	2040489	g__Gellertiella	High	4,165,722	2	Yes	18.43	94.31	0.21
uncultured Ferruginibacter sp.	1042239	g__Ferruginibacter	High	6,524,942	1	Yes	46.49	99.26	0.25
uncultured Mucilaginibacter sp.	797541	g__Mucilaginibacter	High	3,120,558	1	Yes	75.38	97.62	0.00
uncultured Sphingomonas sp.	158754	g__Sphingomonas	High	3,790,002	1	Yes	17.18	98.98	1.19
Bacteroidia bacterium	2044936	g__UBA4416	High	4,615,416	31	No	10.5	98.57	0.95
uncultured Flavobacterium sp.	165435	g__Flavobacterium	High	5,236,199	35	No	8.08	96.57	0.58
uncultured Pelomonas sp.	452440	g__Pelomonas	High	5,892,783	187	Partial	6.23	92.95	4.89

## Methods

### Sample acquisition

The specimen with ID GHC0000127 (ToLID odEunFrag1), collected and identified by Sally Leys, was used for PacBio HiFi sequencing. *Eunapius fragilis* gemmules were collected in January 2022 from a community-run fish hatchery in O’Connor Lake in northern Vancouver Island, British Columbia, Canada. After a period of 6 weeks, allowing vernalisation ( Benfey & Reiswig, 1982) gemmules were removed from the adult tissue and hatched in on sterile Petri dishes in Strekal’s medium following previously described methods ( Leys *et al.*, 2019; Strekal & McDiffett, 1974). Dishes were kept in the dark and media was changed every two days until the sponge tissues formed a lawn covering the dishes. The sterile culture medium and dark conditions produced tissues that were free of algal symbionts, but with its associated microbiome carried over from the adult sponge. The specimen was snap-frozen in liquid nitrogen.

The specimen used for RNA sequencing (specimen ID NSU0023201, ToLID odEunFrag2) was collected by Cole Easson, and identified by Damon Stinson and Cole Easson. *Eunapius fragilis* tissue was collected on 5 November 2020 from Stewart’s Creek in Smyrna, Tennessee, USA. No gemmules were observed during collection, although gemmuloscleres were later observed using microscopy. All sponges were found growing on the downstream side or underside of rocks across the channel of the creek. Using a razor blade, tissue was scraped from the rock and collected tissue was placed in sterile 1.8 mL microcentrifuge tubes and covered with cold 95% ethanol. Samples were placed on ice until they were frozen at –20 °C at Middle Tennessee State University.

### Nucleic acid extraction

The workflow for high molecular weight (HMW) DNA extraction at the Wellcome Sanger Institute (WSI) Tree of Life Core Laboratory includes a sequence of procedures: sample preparation and homogenisation, DNA extraction, fragmentation and purification. Detailed protocols are available on protocols.io ( Denton *et al.*, 2023). The odEunFrag1 sample was prepared for DNA extraction by weighing and dissecting it on dry ice ( Jay *et al.*, 2023). Prior to DNA extraction, the sponge sample was bathed in “L buffer” (10 mM Tris, pH 7.6, 100 mM EDTA, 20 mM NaCl), minced into small pieces using a scalpel and the cellular interior separated from the mesohyl using forceps ( Lopez, 2022). HMW DNA was extracted using the Automated MagAttract v2 protocol ( Oatley *et al.*, 2023a). For ULI PacBio sequencing, DNA was fragmented using the Covaris g-TUBE method ( Oatley *et al.*, 2023c). Sheared DNA was purified by solid-phase reversible immobilisation, using AMPure PB beads to eliminate shorter fragments and concentrate the DNA ( Oatley *et al.*, 2023b). The concentration of the sheared and purified DNA was assessed using a Nanodrop spectrophotometer and Qubit Fluorometer using the Qubit dsDNA High Sensitivity Assay kit. Fragment size distribution was evaluated by running the sample on the FemtoPulse system.

RNA was extracted from tissue of odEunFrag2 in the Tree of Life Laboratory at the WSI using the RNA Extraction: Automated MagMax™ *mir*Vana protocol ( do Amaral *et al.*, 2023). The RNA concentration was assessed using a Nanodrop spectrophotometer and a Qubit Fluorometer using the Qubit RNA Broad-Range Assay kit. Analysis of the integrity of the RNA was done using the Agilent RNA 6000 Pico Kit and Eukaryotic Total RNA assay.

### Sequencing

Library preparation and sequencing was performed at the WSI Scientific Operations core. Pacific Biosciences HiFi circular consensus DNA sequencing libraries were constructed according to the manufacturers’ instructions for ULI sequencing. Samples were sequenced on a Revio and Sequel IIe instruments (Pacific Biosciences, California, USA). Hi-C data were also generated from tissue of odEunFrag1 using the Arima2 kit and sequenced on the Illumina NovaSeq 6000 instrument. Poly(A) RNA-Seq libraries were constructed using the NEB Ultra II RNA Library Prep kit. RNA sequencing was performed on an Illumina NovaSeq 6000 instrument.

### Genome assembly, curation and evaluation

***Assembly***

The HiFi reads were assembled using Hicanu ( Nurk *et al.*, 2020). Haplotypic duplications were identified and removed using purge_dups ( Guan *et al.*, 2020). The Hi-C reads were mapped to the primary contigs using bwa-mem2 ( Vasimuddin *et al.*, 2019). The contigs were further scaffolded using the provided Hi-C data ( Rao *et al.*, 2014) in YaHS ( Zhou *et al.*, 2023) using the --break option for handling potential misassemblies. The scaffolded assemblies were evaluated using Gfastats ( Formenti *et al.*, 2022), BUSCO ( Manni *et al.*, 2021) and MERQURY.FK ( Rhie *et al.*, 2020).

The mitochondrial genome was assembled using MitoHiFi ( Uliano-Silva *et al.*, 2023), which runs MitoFinder ( Allio *et al.*, 2020) and uses these annotations to select the final mitochondrial contig and to ensure the general quality of the sequence.

***Assembly curation***

The assembly was decontaminated using the Assembly Screen for Cobionts and Contaminants (ASCC) pipeline. Flat files and maps used in curation were generated via the TreeVal pipeline ( Pointon *et al.*, 2023). Manual curation was conducted primarily in PretextView ( Harry, 2022) and HiGlass ( Kerpedjiev *et al.*, 2018), with additional insights provided by JBrowse2 ( Diesh *et al.*, 2023). Scaffolds were visually inspected and corrected as described by Howe *et al*. (2021). Any identified contamination, missed joins, and mis-joins were amended, and duplicate sequences were tagged and removed. The curation process is documented at https://gitlab.com/wtsi-grit/rapid-curation.

***Assembly quality assessment***

The Merqury.FK tool ( Rhie *et al.*, 2020), run in a Singularity container ( Kurtzer *et al.*, 2017), was used to evaluate *k*-mer completeness and assembly quality for the primary and alternate haplotypes using *k*-mer databases ( *k* = 31) computed prior to genome assembly. The analysis outputs included assembly QV scores.

A Hi-C contact map was produced for the final version of the assembly. The Hi-C reads were aligned using bwa-mem2 ( Vasimuddin *et al.*, 2019) and the alignment files were combined using SAMtools ( Danecek *et al.*, 2021). The Hi-C alignments were converted into a contact map using BEDTools ( Quinlan & Hall, 2010) and the Cooler tool suite ( Abdennur & Mirny, 2020). The contact map is visualised in HiGlass ( Kerpedjiev *et al.*, 2018).

The blobtoolkit pipeline is a Nextflow port of the previous Snakemake Blobtoolkit pipeline ( Challis *et al.*, 2020). It aligns the PacBio reads in SAMtools and minimap2 ( Li, 2018) and generates coverage tracks for regions of fixed size. In parallel, it queries the GoaT database ( Challis *et al.*, 2023) to identify all matching BUSCO lineages to run BUSCO ( Manni *et al.*, 2021). For the three domain-level BUSCO lineages, the pipeline aligns the BUSCO genes to the UniProt Reference Proteomes database ( Bateman *et al.*, 2023) with DIAMOND blastp ( Buchfink *et al.*, 2021). The genome is also divided into chunks according to the density of the BUSCO genes from the closest taxonomic lineage, and each chunk is aligned to the UniProt Reference Proteomes database using DIAMOND blastx. Genome sequences without a hit are chunked using seqtk and aligned to the NT database with blastn ( Altschul *et al.*, 1990). The blobtools suite combines all these outputs into a blobdir for visualisation.

The blobtoolkit pipeline was developed using nf-core tooling ( Ewels *et al.*, 2020) and MultiQC ( Ewels *et al.*, 2016), relying on the Conda package manager, the Bioconda initiative ( Grüning *et al.*, 2018), the Biocontainers infrastructure ( da Veiga Leprevost *et al.*, 2017), as well as the Docker ( Merkel, 2014) and Singularity ( Kurtzer *et al.*, 2017) containerisation solutions.

Table 5 contains a list of relevant software tool versions and sources.

**Table 5.** Software tools: versions and sources.

Software tool	Version	Source
BEDTools	2.30.0	https://github.com/arq5x/bedtools2
bin3C	0.3.3	https://github.com/cerebis/bin3C
BLAST	2.14.0	ftp://ftp.ncbi.nlm.nih.gov/blast/executables/blast+/
BlobToolKit	4.3.3	https://github.com/blobtoolkit/blobtoolkit
BUSCO	5.5.0	https://gitlab.com/ezlab/busco
bwa-mem2	2.2.1	https://github.com/bwa-mem2/bwa-mem2
CheckM	1.2.1	https://github.com/Ecogenomics/CheckM
Cooler	0.8.11	https://github.com/open2c/cooler
DIAMOND	2.1.8	https://github.com/bbuchfink/diamond
dRep	3.4.0	https://github.com/MrOlm/drep
fasta_windows	0.2.4	https://github.com/tolkit/fasta_windows
FastK	427104ea91c78c3b8b8b49f1a7d6bbeaa869ba1c	https://github.com/thegenemyers/FASTK
Gfastats	1.3.6	https://github.com/vgl-hub/gfastats
GoaT CLI	0.2.5	https://github.com/genomehubs/goat-cli
GTDB-TK	2.3.2	https://github.com/Ecogenomics/GTDBTk
Hicanu	2.2	https://github.com/marbl/canu
HiGlass	44086069ee7d4d3f6f3f0012569789ec138f42b84aa44357826c0b6753eb28de	https://github.com/higlass/higlass
MAGScoT	1.0.0	https://github.com/ikmb/MAGScoT
MaxBin	2.7	https://sourceforge.net/projects/maxbin/
MerquryFK	d00d98157618f4e8d1a9190026b19b471055b22e	https://github.com/thegenemyers/MERQURY.FK
MetaBat2	2.15-15-gd6ea400	https://bitbucket.org/berkeleylab/metabat/src/master/
metaMDBG	Pre-release	https://github.com/GaetanBenoitDev/metaMDBG
MetaTOR	Pre-release	https://github.com/koszullab/metaTOR
Minimap2	2.24-r1122	https://github.com/lh3/minimap2
MitoHiFi	2	https://github.com/marcelauliano/MitoHiFi
MultiQC	1.14, 1.17, and 1.18	https://github.com/MultiQC/MultiQC
Nextflow	23.04.1	https://github.com/nextflow-io/nextflow
PretextView	0.2	https://github.com/sanger-tol/PretextView
PROKKA	1.14.5	https://github.com/vdejager/prokka
purge_dups	1.2.3	https://github.com/dfguan/purge_dups
samtools	1.18	https://github.com/samtools/samtools
sanger-tol/ascc	-	https://github.com/sanger-tol/ascc
sanger-tol/blobtoolkit	0.3.0	https://github.com/sanger-tol/blobtoolkit
Seqtk	1.3	https://github.com/lh3/seqtk
Singularity	3.9.0	https://github.com/sylabs/singularity
TreeVal	1.2.0	https://github.com/sanger-tol/treeval
YaHS	1.1a.2	https://github.com/c-zhou/yahs

***Metagenome assembly***

The metagenome assembly was generated using metaMDBG ( Benoit *et al.*, 2024) and binned using MetaBAT2 ( Kang *et al.*, 2019), MaxBin ( Wu *et al.*, 2014), bin3C ( DeMaere & Darling, 2019), and MetaTOR. The resulting bin sets of each binning algorithm were optimised and refined using MAGScoT ( Rühlemann *et al.*, 2022). PROKKA ( Seemann, 2014) was used to identify tRNAs and rRNAs in each bin, CheckM ( Parks *et al.*, 2015) (checkM_DB release 2015-01-16) was used to assess bin completeness/contamination, and GTDB-TK ( Chaumeil *et al.*, 2022) (GTDB release 214) was used to taxonomically classify bins. Taxonomic replicate bins were identified using dRep ( Olm *et al.*, 2017) with default settings (95% ANI threshold). The final bin set was filtered for bacteria and archaea. All bins were assessed for quality and categorised as metagenome-assembled genomes (MAGs) if they met the following criteria: contamination ≤ 5%, presence of 5S, 16S, and 23S rRNA genes, at least 18 unique tRNAs, and either ≥ 90% completeness or ≥ 50% completeness with fully circularised chromosomes. Bins that did not meet these thresholds, or were identified as taxonomic replicates of MAGs, were retained as ‘binned metagenomes’ provided they had ≥ 50% completeness and ≤ 10% contamination. A cladogram based on NCBI taxonomic assignments was generated using the ‘taxonomizr’ package in R. The tree was visualised and annotated using iTOL ( Letunic & Bork, 2024). Software tool versions and sources are given in Table 4.

### Genome annotation

The Ensembl Genebuild annotation system ( Aken *et al.*, 2016) was used to generate annotation for the *Eunapius fragilis* assembly (GCA_963681505.1) in Ensembl Rapid Release at the EBI. Annotation was created primarily through alignment of transcriptomic data to the genome, with gap filling via protein-to-genome alignments of a select set of proteins from UniProt ( UniProt Consortium, 2019).

### Wellcome Sanger Institute – Legal and Governance

The materials that have contributed to this genome note have been supplied by a Darwin Tree of Life Partner. The submission of materials by a Darwin Tree of Life Partner is subject to the **‘Darwin Tree of Life Project Sampling Code of Practice’**, which can be found in full on the Darwin Tree of Life website here. By agreeing with and signing up to the Sampling Code of Practice, the Darwin Tree of Life Partner agrees they will meet the legal and ethical requirements and standards set out within this document in respect of all samples acquired for, and supplied to, the Darwin Tree of Life Project. 

Further, the Wellcome Sanger Institute employs a process whereby due diligence is carried out proportionate to the nature of the materials themselves, and the circumstances under which they have been/are to be collected and provided for use. The purpose of this is to address and mitigate any potential legal and/or ethical implications of receipt and use of the materials as part of the research project, and to ensure that in doing so we align with best practice wherever possible. The overarching areas of consideration are:

•   Ethical review of provenance and sourcing of the material

•   Legality of collection, transfer and use (national and international)

Each transfer of samples is further undertaken according to a Research Collaboration Agreement or Material Transfer Agreement entered into by the Darwin Tree of Life Partner, Genome Research Limited (operating as the Wellcome Sanger Institute), and in some circumstances other Darwin Tree of Life collaborators.

## Data Availability

European Nucleotide Archive: Eunapius fragilis. Accession number PRJEB63655; https://identifiers.org/ena.embl/PRJEB63655. The genome sequence is released openly for reuse. The *Eunapius fragilis* genome sequencing initiative is part of the Aquatic Symbiosis Genomics (ASG) project ( https://www.ebi.ac.uk/ena/browser/view/PRJEB43743). All raw sequence data and the assembly have been deposited in INSDC databases. Raw data and assembly accession identifiers are reported in Table 1 and Table 2.

## Author information

Members of the Wellcome Sanger Institute Tree of Life Management, Samples and Laboratory Team are listed here: https://doi.org/10.5281/zenodo.10066175.

Members of the Wellcome Sanger Institute Scientific Operations: Sequencing Operations are listed here: https://doi.org/10.5281/zenodo.10043364.

Members of the Wellcome Sanger Institute Tree of Life Core Informatics team are listed here: https://doi.org/10.5281/zenodo.10066637.

Members of the European Bioinformatics Institute ASG Data Portal team are listed here: https://doi.org//10.5281/zenodo.10076466.

Members of the Wellcome Sanger Institute/Aquatic Symbiosis Genomics Project Leadership are listed here: https://doi.org/10.5281/zenodo.10184833.
