# Supplementary material for: The chromosomal genome sequence of the fragile freshwater sponge, Eunapius fragilis (Leidy, 1851) and its associated microbial metagenome sequences
Source: Wellcome Open Res. 2025 May 23;10:268. [Version 1] doi: 10.12688/wellcomeopenres.24165.1 (PMC12616128; doi:10.12688/wellcomeopenres.24165.1)
Supplement: Peer review history

# Reviewer report: Reviewer response for version 1

**DOI:** 10.21956/wellcomeopenres.26656.r138705

**Published:** 2025-11-13

**Authors:** Jayan Duminda M Senevirathna

**Affiliations:** 1 Uva Wellassa University, Badulla, Uva Province, Sri Lanka

The article is well-written and logically reported. Raw data is available and reproducible. However, the following are for more clarification;

 The reported BUSCO completeness (60%) appears relatively low for a chromosome-level assembly. Typically, chromosome-level assemblies exhibit BUSCO completeness above 90%, reflecting a high level of genome completeness and contiguity.

 Please clarify how chromosome identification was achieved with this level of completeness. It would be useful to describe the methods used for chromosome anchoring (e.g., Hi-C, optical mapping) and justify the reliability of the assembly given the BUSCO results.

Are sufficient details of methods and materials provided to allow replication by others?

Yes

Is the rationale for creating the dataset(s) clearly described?

Yes

Are the datasets clearly presented in a useable and accessible format?

Yes

Are the protocols appropriate and is the work technically sound?

Yes

Reviewer Expertise:

Aquatic Biotechnology and Genetics

I confirm that I have read this submission and believe that I have an appropriate level of expertise to confirm that it is of an acceptable scientific standard.

# Reviewer report: Reviewer response for version 1

**DOI:** 10.21956/wellcomeopenres.26656.r124286

**Published:** 2025-06-14

**Authors:** Emily C Giles

**Affiliations:** 1 Universidad Austral de Chile, Valdivia, Los Ríos Region, Chile

This manuscript presents the genome assembly and annotation of Eunapis fragilis. The authors include MAGs assembled from the metagenome as well as the mitochondrial assembly and annotation. The genome is assembled into 23 pseudochromosomes that house over 99% of the assembly. This is a great assembly and resource for those working on marine genomics. My only questions include the following:

 Was the same tissue sample used for the PacBio sequencing and the HiC map? This is not clearly stated and I believe different biosample numbers are given.

 Furthermore, it is not clear why this genome report includes the Ensembl annotation rather than the ab initio protein model. It is great that the authors provide a link to the ab initio model, but why is this not included as the main annotation in this report? It is noted that the RNAseq data were derived from a different specimen than that used for the assembly, perhaps this is the reason?

 It would also be great if the authors could comment on the low BUSCO scores recovered. Complete buscos are only around 60%. Did the authors attempt to manually blast any of the complete buscos against their assembly to see if they are actually present with less stringent search parameters? Is this genome particularly depauperate in genes?

 Overall, it was great to read a genome report with well detailed methods that did not require the reader to reference multiple linked github pages.

Are sufficient details of methods and materials provided to allow replication by others?

Yes

Is the rationale for creating the dataset(s) clearly described?

Yes

Are the datasets clearly presented in a useable and accessible format?

Yes

Are the protocols appropriate and is the work technically sound?

Yes

Reviewer Expertise:

Comparative genomics, population genomics, speciation, biosecurity, marine ecosystems

I confirm that I have read this submission and believe that I have an appropriate level of expertise to confirm that it is of an acceptable scientific standard.
